# Health and suffering are associated with social support: a cross-sectional study of women and mothers with exhaustion and pain

**DOI:** 10.1186/s12905-021-01398-y

**Published:** 2021-06-26

**Authors:** Anja Gebhardt, Ann Langius-Eklöf, Susanne Andermo, Maria Arman

**Affiliations:** 1 Division of Nursing, Department of Neurobiology, Care Sciences and Society, Karolinska Institutet, 14183 Huddinge, Sweden; 2 Department of Global Public Health, Karolinska Institutet, 17165 Solna, Sweden

**Keywords:** Caring science, Nursing theory, Existential health, Chronic pain, Burnout, Female, Parents

## Abstract

**Background:**

Despite women are generally overrepresented in behavioral, mental, and musculoskeletal disorders, motherhood as a central part of women’s life is poorly understood in relation to exhaustion and long-lasting pain. Mothers’ health impairments imply suffering both for herself and her family. A profound understanding of health is needed taking mothers’ subjective health experience, their suffering and life situation into account to give women, their families and society better prerequisites to alleviate exhaustion and long-lasting pain. The aim of the study was to describe health and suffering of women and mothers undergoing rehabilitation for long-lasting pain and exhaustion and its correlation with perceived social support.

**Methods:**

The study had a cross-sectional design with an exploratory approach. A main sample consisted of 166 women undergoing rehabilitation for exhaustion and long-lasting pain and a reference sample included 129 women working and studying within health care professions. Both samples included women with and without children. Women’s subjective health and suffering was assessed from a caring science perspective using the recently developed and validated Health and Suffering Scale. Two additional scales measuring exhaustion and social support were distributed among the two samples. Descriptive statistics and multiple linear regression models, including health and suffering and perceived social support, were analyzed.

**Results:**

Mothers undergoing rehabilitation for pain and exhaustion reported significantly poorer health and more suffering compared to healthy mothers, but similar health and suffering when compared with childless women in rehabilitation. Health and suffering were correlated with perceived social support among both healthy and exhausted mothers. In both samples, the correlation between health and suffering and social support was stronger among mothers than among women without children.

**Conclusions:**

Women and mothers living with exhaustion and long-lasting pain show signs of unbearable suffering and perceived insufficient social support. Social support from various sources particularly helps mothers to create meaning in life and make their suffering bearable. Hence, health care must address the fact that mothers are dependent on their immediate social environment and that this dependency interacts with their health and suffering on an existential level.

## Background

Women are generally overrepresented in behavioral, mental, and musculoskeletal disorders [1–4]. In Sweden, particularly women aged between 30 and 44 years are at risk of developing stress-related illness and exhaustion seemingly related to a double burden of family and working life (throughout this article the term exhaustion is preferred as a broader concept of burnout) [4, 5]. Motherhood is a life-long commitment that continuously influences women’s health even when children have reached adulthood [6–8]. Further, mental health problems during child-rearing years were found to predict exhaustion among employed mothers later in life [9]. In particular women with long-lasting pain, but also individuals with exhaustion, often feel not taken seriously and even disbelieved by the health care system causing them further suffering [10–12]. The significance of suffering has been identified as missing in traditional medicine’s understanding of health [13, 14]. A definition of modest—instead of perfect—health focusing on a human being’s ability to adapt has been called for [13]. Thus, a more profound understanding of health is needed taking a woman’s subjective health experience, including her suffering and life situation into account to give women, their families and society better prerequisites to alleviate and treat exhaustion and long-lasting pain. In caring science, suffering is a basic concept that is considered central to understanding subjective health [15, 16]. This idea of health, building a balanced entity with suffering, moves beyond the definition of perfect health and physical, mental, behavioral symptomatology.

According to nursing theorists and philosophers, the experience of suffering is a natural part of human life affecting a person on an existential level [16–20]. Hence, in caring science, health has been described as building a balanced entity with suffering [16]. Therefore, the experiences of health and suffering are not regarded as mutually exclusive, rather health reflects an individual’s ability to meaningfully integrate suffering into their own life [16]. It has been theorized that meaning-creation with others helps to integrate suffering and to make it bearable [21]. Andermo et al. [15] further conceptualized the theory of health and suffering by developing a health measurement scale that includes following existential prerequisites for a balanced health: to have an understanding of life, to be in a process of development, to perceive hope and meaning in life and to engage in life. The scale estimates the movement between these conditions and their counterpoints, reflecting how well health and suffering are integrated in an individual’s life [15]. Existing literature on the health of mothers mainly applies psychological [22, 23], medical [7, 24], sociological [5, 25] and epidemiological [26] perspectives, but exploration of mothers’ subjective experience acknowledging both their health and their suffering is limited. Investigating mothers’ health and suffering will have implications for the formation and further development of care and rehabilitation of women with exhaustion and pain, but also for the development and implementation of preventive measures. Hence, in this study, subjective health is measured in relation to suffering as conceptualized by Andermo et al. [15].

A woman’s health and well-being are dependent on a country’s policy context and her immediate social context within which she lives as a mother [6, 27, 28]. The positive association between health and social support is well-known and has been studied for decades [29, 30], also in relation to motherhood [27]. However, it has barely been explored among mothers living with exhaustion and long-lasting pain [31, 32]. Such an explorative investigation is of particular interest for three interplaying reasons. First, exhaustion and long-lasting pain are associated with stress [33, 34], second, motherhood is demanding and can be stressful [6, 35, 36] and third, social support is known to have buffering effects on stress [29, 37, 38].

A qualitative study on women living with pain [11] revealed their health and suffering to be intertwined with an overwhelming life. The women cared for the welfare of their environment, including their family and children leaving them exhausted without the care and support they themselves would have needed [11]. Other studies found that mothers suffering from long-lasting pain or exhaustion perceived a lack of social support [31, 32]. Luthar [35] emphasized how in particular stressed mothers need to be nurtured in warm and supportive relationships. Against the background of these studies, we focus on perceived social support as conceptualized by Zimet et al. [39] to investigate how well mothers are embedded in meaningful and nurturing relationships versus being left alone with their desires, needs and problems. Social support is often categorized into three different areas: emotional, instrumental, and informative support. In short, emotional support means to care for someone, whereas instrumental support refers to practical help and informative support means to give advice [29]. Zimet’s scale of perceived social support focuses on emotional and instrumental support [39, 40].

The aim of the study was to describe health and suffering of women and mothers undergoing rehabilitation for long-lasting pain and exhaustion and its correlation with perceived social support.

## Methods

### Design

The study has a cross-sectional design and adopts an exploratively oriented approach where motherhood provides context rather than serving as a discerning variable.

### Participants

Two samples of women were recruited, one patient and one reference sample. The patient sample was consecutively included at a rehabilitation clinic for exhaustion and long-lasting pain in Stockholm, Sweden. There were 297 eligible patients and the response rate after two reminders was 55.9%, yielding 166 participants. The reference sample consisted of nurses, occupational therapists and physiotherapists studying health care programs for specialization within their profession at Karolinska Institutet in Stockholm, Sweden. Students on a specialization level were chosen to match as far as possible the age, profession, and midlife situation of the patient sample. There were 209 eligible students, inclusion was consecutive. The response rate after two reminders was 61.7%, yielding 129 participants.

### Procedure

Patients were informed about the study in the middle of the 10-week rehabilitation period and students were informed at the beginning or the end of a lecture. Patients and students voluntarily signed up for an invitation letter by email that informed them about study participation and that included a personal link to the study’s web survey. Two reminders were sent out after two and four weeks following the first letter. The web survey was identical for all three samples, consisting of sociodemographic questions, the Health and Suffering Scale (HSS), the Karolinska Exhaustion Disorder Scale (KEDS) and the Multidimensional Scale of Perceived Social Support (MSPSS). Data were collected between October 2018 and August 2019.

### Measurements

Sociodemographic data were collected using items from the government agency, *Statistics Sweden* [41]. One study-specific item with a free-comment field collected information about caregiving responsibility for relatives other than children.

The Health and Suffering Scale (HSS) is a self-report scale that measures perceived suffering in relation to health from a caring science perspective [42]. The scale consists of nine items with a 101-step VAS response scale that is placed between word pairs reflecting health and suffering inspired by the theory of Eriksson (2006), for example “life is a struggle – life is a gift”, “life without meaning – meaningful life”, “stuck in negative life patterns – in a process of development”, or “unbearable suffering – bearable suffering”. A low score on an item reflects perceived unbearable suffering and health hindrances in life whereas a high score on an item reflects bearable suffering and the experience of health in life. In the current study, the 101-step VAS scale was transformed to a five-point scale in accordance with a psychometric evaluation of the scale [42]. Possible sum scores range between nine and 45. The nine-item scale was found to have robust psychometric properties among Swedish samples of healthy women and women with exhaustion and long-lasting pain. The scale showed to be internally consistent among mothers in rehabilitation α = 0.94 (CI 0.92–0.95), women without children in rehabilitation α = 0.94 (CI 0.91–0.96), and α = 0.92 (CI 0.93–0.95) among healthy mothers.

The Karolinska Exhaustion Disorder Scale (KEDS) is a self-report measure for exhaustion disorder, validated in Swedish among both healthy and exhausted individuals [43]. KEDS consists of nine items measuring the degree of stress-induced exhaustion through to self-reported ability to concentrate, memory, physical stamina, mental stamina, recovery, sleep, sensory impressions, experience of demands and irritation and anger on a seven-point Likert scale. A high sum score indicates a high degree of exhaustion. Possible scores range from between nine and 63. In the current samples, the internal consistency of the KEDS was α = 0.83 (CI 0.79–0.87) for mothers in rehabilitation, α = 0.87 (CI 0.81–0.92) for women without children in rehabilitation, and α = 0.88 (CI 0.85–0.9) for healthy mothers.

The Multidimensional Scale of Perceived Social Support (MSPSS) was developed by Zimet et al. [39] and translated and culturally adapted to the Swedish context by Ekbäck, Benzein, Lindberg and Årestedt [40]. The MSPSS is a self-report scale that measures perceived social support on a seven-point Likert scale. It consists of 12 items that are evenly distributed across the three dimensions with four items each: family, friends, and significant others. A total sum score of all items ranges from between 12 and 84, sum scores of the subscales range from between four and 28, higher scores indicate higher perceived social support. The Swedish version showed to have robust psychometric properties among women with hirsutism and among nursing students [40]. In the current study, the internal consistency of the MSPSS was α = 0.94 (CI 0.93–0.95) for mothers in rehabilitation, α = 0.95 (CI 0.93–0.97) for women without children in rehabilitation, and α = 0.95 (CI 0.93–0.95) for healthy mothers.

### Statistical analysis

A common approach in exploring factors that possibly influence the health of parents is to compare parents to individuals without children [44, 45]. Thus, estimates of health and suffering and perceived social support among mothers undergoing rehabilitation for long-lasting pain and exhaustion, among women without children in rehabilitation, and among healthy mothers were calculated and described. Two sample t-test was used to compare estimates of health and suffering and perceived social support between mothers and women without children and between mothers in rehabilitation and healthy mothers. Further, the degree of exhaustion was compared to explore possible differences in exhaustion between mothers in different rehabilitation programs as well as healthy mothers.

Linear regression was performed to investigate whether perceived social support accounts for a variation in health and suffering among women. In the main regression model, the total sum score of the Health and Suffering Scale was included as a dependent variable and perceived social support as measured by the total sum score of MSPSS was included as a single independent variable. In a subsequent model the subscales were included as separated independent variables in a multiple regression model to explore their specific association with health and suffering, adjusted for the remaining other two sources of support.

In a last step, based only on data of mothers, the main model was extended to multiple linear regression models with adjustments for possible confounders as follows: degree of exhaustion disorder (KEDS score), living in a relationship (cohabiting/single coded as + 0.5/− 0.5), number of children living at home and age. KEDS was included to adjust for the association between exhaustion and suffering and its possible negative effect on social support (exhausted and suffering mothers might not make use of their social environment). The other adjustments were of theoretical interest because living in a relationship is assumed to be related to perceived social support, the number of children living at home can affect mothers’ health [4] and possibly mothers’ dependence on social support. Finally, the results of the regression models were compared on a structural level between the two groups of mothers.

The following assumptions for linear regression analyses were fulfilled. The sample size was with good margins above the minimum requirements of variable to participant ratio of 1:10 [46]. The residuals in the applied models were normally distributed and homoscedastic. The residuals in the extended model indicated a trend towards heteroscedasticity when applied among healthy mothers, no such indications were observed for the model among mothers in rehabilitation. Influence of any observations on the multiple regression models as calculated by Cook’s distance had maximum values of < 0.23. Collinearity diagnostics according to Belsley [47] indicated acceptable multicollinearity for the model, including all three subscales of MSPSS as separated independent variables. In the extended multiple regression model, any indications of dependency of the variates were not found.

Analyses were run in MatLab version R2019b (MathWorks, Massachusetts, USA). Cohen’s *d* was calculated according to Fritz, Morris and Richler [48] using the MatLab code by Bettinardi [49].

### Missing items

Among women in rehabilitation, 9.8% of HSS data were missing versus 1.3% among healthy mothers. In the current study, HSS data were generated by a digital slider which by default was positioned on zero, implying unbearable suffering. For some participants, this default position might have appeared to be in the correct position of unbearable suffering without having moved the slider. In contrast, data in MSPSS and KEDS were missing for 0.8% and 1.4% of observations among women in rehabilitation versus 0% and 0.1% among healthy mothers. Missing values were assumed to have an equal distribution among all three instruments and were therefore substituted with a value of zero in the HSS generated data.

## Results

### Characteristics of the sample

The majority of women (77.7%, n = 129) in rehabilitation had children at any age (Table 1). Seventy-five mothers (58.1%) were undergoing rehabilitation for long-lasting pain, 51 mothers (39.1%) were undergoing rehabilitation for exhaustion (Table 2). Among the healthy women, 108 (83.7%) were mothers of children at any age. Eight mothers (6.2%) from the healthy group reported having received rehabilitation for long-lasting pain during the last three months (Table 2).

**Table 1:** Sociodemographic characteristics of respondents

	Mothers with exhaustion and pain	Women without children with exhaustion and pain	Healthy mothers	Healthy women without children
	N = 129 n (%)	N = 37 n (%)	N = 108 n (%)	N = 21 n (%)
Age, years, mean +/− SD	50.1 +/− 9.7	39.3 +/− 11.7	39.4 +/− 7.3	31.7 +/− 6.6
Marital status				
Single	37 (28.7%)	21 (56.8%)	13 (12.0%)	13(61.9%)
Married/cohabiting	92 (71.3%)	16 (43.2%)	95 (88.0%)	8 (38.1%)
Caring responsibility for relative/s	17 (13.2%)	5 (13.5%)	3 (2.8%)	1 (4.8%)
Education				
Comprehensive School	9 (7.0%)	2 (5.4%)	0 (0%)	0
Secondary School	45 (34.9%)	14 (37.8%)	0 (0%)	0
Higher education/University	75 (58.1%)	21 (56.8%)	108 (100%)	21 (100%)
Total household income (Swedish crowns/month)				
Under 15t SEK	15 (11.6%)	4 (10.8%)	1 (0.9%)	0
Under 30t SEK	21 (16.3%)	12 (32.4%)	4 (3.7%)	2 (9.5%)
Under 45t SEK	29 (22.5%)	14 (37.8%)	28 (25.9%)	4 (19.0%)
Under 60t SEK	25 (19.4%)	6 (16.2%)	28 (25.9%)	11 (52.4%)
Over 60t SEK	39 (30.2%)	1 (2.7%)	47 (43.5%)	4 (19.0%)
Employment status				
Employed	64 (49.6%)	9 (24.3%)	96 (88.9%)	19 (90.5%)
Self-employed	9 (7.0%)	2 (5.4%)	2 (1.9%)	0
Student	1 (0.8%)	4 (10.8%)	102 (94.4%)	21 (100%)
Retired	12 (9.3%)	2 (5.4%)	1 (0.9%)	0
Off-duty/parental leave	2 (1.6%)	0 (0%)	17 (15.7%)	4 (19.0)
Job seeking	5 (3.9%)	7 (18.9%)	0 (0%)	0
Home worker	7 (5.4%)	6 (16.2%)	5 (4.6%)	0
Other/Nothing	4 (3.1%)	1 (2.7%)	0 (0%)	0
Country of birth				
Sweden	106 (82.2%)	36 (97.3%)	94 (87.0%)	18 (85.7%)
Scandinavia	4 (3.1%)	1 (2.7%)	1 (0.9%)	0
Europe	10 (7.8%)	0 (%)	2 (1.9%)	1 (4.8%)
Outside Europe	9 (7.0%)	0 (%)	11 (10.2%)	2 (9.5%)

**Table 2:** Sick leave and rehabilitation among respondents

	Mothers living with exhaustion and pain	Women without children living with exhaustion and pain	Healthy mothers	Healthy women without children
	n = 129	n = 37	n = 108	n = 21
	n (%)	n (%)	n (%)	n (%)
Long-term sick leave	76 (59.0%)	23 (62.2%)	0 (0%)	0
Rehabilitation				
Long-lasting pain	75 (58.1%)	19 (51.4%)	8 (7.4%)	0
Exhaustion	51 (39.5%)	18 (48.6%)	0 (0%)	0
Other/no rehabilitation	3 (2.3%)	0 (0%)	100 (92.6%)	0

Mothers undergoing rehabilitation were older and had to a larger extent older children living at home when compared with healthy mothers (Tables 1, 3). Seventeen mothers (13.2%) in rehabilitation versus three mothers (2.8%) in the healthy sample reported having a caring responsibility for other relatives, e.g., a frail parent. An additional five (3.9%) women in rehabilitation versus two healthy mothers (1.9%) informed about having caregiving responsibility for their children with special needs, e.g., Down syndrome or autism. Almost a third of mothers in rehabilitation (28.7%) were single mothers whereas 12% of the healthy mothers reported living without a partner. Most women without children were living without a partner in both samples. These characteristics are summarized in Table 1.

**Table 3:** Characteristics of children living at home

	Mothers in rehabilitation	Healthy mothers
	(n = 129)	(n = 108)
Participants with		
Small children, n (%)	25 (19.4%)	57 (52.8%)
Schoolers, n (%)	41 (31.8%)	57 (52.8%)
Adolescents, n (%)	34 (26.4%)	13 (12%)
Adult children, n (%)	29 (22.5%)	14 (13%)
Children not living at home	41 (31.8%)	5 (4.6%)
Number of children per participant		
Number of children living at home, mean (min–max)	2.0 (1–8)	2.1 (1–4)
Small children^a^, mean (min–max)	0.42 (0–4)	0.78 (0–3)
Schoolers^b^, mean (min–max)	0.65 (0–6)	0.83 (0–3)
Adolescents^c^, mean (min–max)	0.47 (0–2)	0.37 (0–3)
Adult^d^, mean (min–max)	0.48 (0–3)	0.14 (0–1)

^a^0–5 years; ^b^6–12 years; ^c^13–17 years; ^d^18 years and older

There was no significant difference in the degree of exhaustion disorder (KEDS sum score mean +/ − SD) between mothers undergoing rehabilitation for pain (41.9 +/ − 7.8) and mothers undergoing rehabilitation for exhaustion (41.1 +/− 8.1). Mothers in rehabilitation reported significantly higher KEDS scores (41.6 +/− 7.8) than healthy mothers (25.3 +/− 8.7; *p* < 0.05, *d* = 2.1).

### Health and suffering and perceived social support

Health and suffering as measured by HSS and perceived social support as measured by MSPSS reported by all four sub samples are presented in Table 4. Mothers undergoing rehabilitation for pain and exhaustion reported significantly lower estimates of HSS than healthy mothers (*p* < 0.001, *d* = 1.518), but similar estimates of HSS when compared with childless women in rehabilitation. Healthy mothers on the other hand revealed to have significantly higher HSS scores than their childless healthy counterparts (*p* = 0.012, *d* = 0.608).

**Table 4:** Health and suffering and perceived social support among mothers living with exhaustion and long-lasting pain compared to healthy mothers and women without children

	Mothers		Women without children	
	With exhaustion and pain	Healthy	With exhaustion and pain	Healthy
N = 295	n = 129	n = 108^a^	n = 37	n = 21
HSS mean +/− SD	26.5 +/− 8.2***	37.9 +/− 6.5*	26.3 +/− 8.9	33.8 +/− 7.5
MSPSS (total score) mean +/− SD	56.8 +/− 15.6***	68.7 (13.2)	58.6 +/− 16.2	66.7 +/− 11.8
Subscales MSPSS				
Family mean +/− SD	18.5 +/− 6.2***	22.6 +/− 5.3	19.0 +/− 7.1	21.0 +/− 5.5
Friends mean +/− SD	17.2 +/− 5.8***	21.5 +/− 5.2	17.8 +/− 5.3	21.1 +/− 6.1
Significant others Mean +/− SD	21.1 +/− 6.2***	24.6 +/− 4.6	21.8 +/− 6.1	24.6 +/− 3.6

*HSS* Health and Suffering Scale, *MSPSS* Multidimensional Scale of Perceived Social Support

^***^Significantly (*p* < 0.001) different to healthy mothers, two sample t-test. *significantly different (*p* = 0.012) to healthy women without children, two sample t-test. ^a^Eight mothers having received rehabilitation for pain during the last three months were included

MSPSS scores differed significantly between mothers in rehabilitation and healthy mothers (*p* < 0.001, *d* = 0.82), but not between mothers and childless women in rehabilitation. This significant difference of MSPSS between the two groups of mothers was maintained when splitting the general measure of MSPSS into its three subscales of ‘family’, ‘friends’, and ‘significant others’ (*p* < 0.001, *d* = 0.636– 0.774) (Table 4). There were no significant differences in any estimates of MSPSS between healthy mothers and healthy women without children.

### Health and suffering in relation to perceived social support among women in rehabilitation

In the main regression model (Table 5), the association between HSS (sum score) and MSPSS (total sum score) was explored. For women without children, the model explained 25.3% (F_2,35_ = 11.9, *p* < 0.05) of the variability of HSS. The model explained 34.3% (*F*_2,127_ = 66.3, *p* < 0.05) of the variability of HSS estimates among mothers undergoing rehabilitation. MSPSS was found to be significantly correlated with HSS (b = 0.31, *p* < 0.05). Thus, the higher the levels of social support reported, the better health and the less suffering the mothers perceived.

**Table 5:** Linear regression models with health and suffering (HSS) as the criterion variable among women in rehabilitation and among healthy women

	Mothers in rehabilitation (n = 129)			Childless women in rehabilitation (n = 37)			Healthy mothers (n = 108)			Healthy childless women (n = 21)		
	*b*	*SE (b)*	β	*b*	*SE (b)*	β	*b*	*SE (b)*	β	*b*	*SE (b)*	β
Main model with MSPSS total sum score as single independent variable												
Constant	8.89	2.24	-	10.173	4.842	-	20.55	2.889	-	*17.184*	*9.169*	-
MSPSS (total sum score)	0.31***	0.038	0.586	0.275**	0.08	0.453	0.252***	0.041	0.5099	0.249	0.135	0.389
R^2^ (R^2^_adj_)	0.343 (0.338)			0.253 (0.232)			0.26 (0.253)			0.151 (0.107)		

*HSS* Health and Suffering Scale, *MSPSS* Multidimensional Scale of Perceived Social Support; b, unstandardized coefficient; β, standardized coefficient; ***p < 0.001; **p < 0.01

In the multiple linear regression model in which all three MSPSS subscales were included as separate independent variables (*F*_4,125_ = 22.5, *p* < 0.001; R^2^ = 0.351), the subscale ‘significant others’ had the strongest correlation with HSS of all three (b = 0.487, *p* = 0.002). The subscale ‘family’ was found to be non-significant (Table 6).

**Table 6:** Multiple linear regression models with health and suffering (HSS) as the criterion variable among mothers in rehabilitation and among healthy mothers

	Health and suffering among mothers in rehabilitation (n = 129)			Health and suffering among healthy mothers (n = 108)		
	*b*	*SE (b)*	β	*b*	*SE (b)*	β
Regression models with MSPSS subscales as separated independent variables						
Constant	8.667	2.254	–	19.921	2.965	–
* Family*	0.164	0.14	0.1235	0.38*	0.159	0.3111
* Friends*	0.263*	0.126	0.1848	− 0.062	0.128	− 0.0503
* Significant others*	0.487***	0.155	0.3661	0.435*	0.197	0.3059
R^2^ (R^2^_adj_)	0.351 (0.335)			0.305 (0.285)		
*Regression models adjusted for possible confounders*						
Constant	37.382	6.056	–	30.197	6.022	–
MSPSS (total sum score)	0.21***	0.037	0.3960	0.205***	0.047	0.4153
KEDS (total sum score)	− 0.462***	0.072	− 0.4365	− 0.185*	0.07	− 0.2485
Living in a relationship (cohabiting/single coded as + 0.5/ − 0.5)	1.903	1.215	0.1048	− 1.202	1.729	− 0.0604
Number of children living at home	− 0.273	0.458	− 0.0438	− 0.14	0.639	− 0.0187
Age (participant)	− 0.072	0.064	− 0.0844	− 0.026	0.076	− 0.0292
R^2^ (R^2^_adj_)	0.525 (0.505)	0.313 (0.279)				

*HSS* Health and Suffering Scale, *MSPSS* Multidimensional Scale of Perceived Social Support, *KEDS* Karolinska Exhaustion Disorder Scale; b, unstandardized coefficient; β, standardized coefficient; *p < 0.05; ***p < 0.001

In a last step, the correlation of MSPSS with HSS was adjusted for KEDS (sum score), living in a relationship, number of children living at home and age of the mothers. This extended model (*F*_6,123_ = 27.2, *p* < 0.001) explained 52.5% of the variability of HSS scores among mothers. MSPSS remained significantly correlated (b = 0.21, *p* < 0.001) with HSS when adjusted for KEDS, living in a relationship, number of children at home and mothers’ age. KEDS was found to have a significant inverse correlation with HSS, implying that mothers experienced poorer health and more suffering with increasing degrees of exhaustion (b =  − 0.462, *p* < 0.001). Remaining independent variables were found to be non-significant (Table 6).

### Health and suffering in relation to perceived social support among healthy women

The regression analyses of the data of healthy mothers (*F*_2,106_ = 37.2, *p* < 0.001) mainly reflected a similar result pattern as obtained in the rehabilitation sample with MSPSS being significantly correlated with HSS in all models (b = 0.252, *p* < 0.001), but with less variability explained compared to the rehabilitation sample. This difference was specifically evident for the extended model that explained 20.9% less variance of HSS among healthy mothers compared to mothers in rehabilitation. The main model explained 15.1% of the variability of HSS estimates among healthy women without children, but was non-significant (*F*_2,19_ = 3.38, *p* > 0.05) (Table 5).

There was one more exception from the general result pattern. In the multiple linear regression model with all three MSPSS subscales included as separate independent variables (F_4,104_ = 15.2, *p* < 0.001; R^2^ = 0.305), the MSPSS subscale ‘friends’ was non-significant. In similarity with the results among mothers in rehabilitation, MSPSS ‘significant others’ had the strongest correlation (b = 0.435, *p* = 0.029) with HSS among healthy mothers. In contrast to mothers in rehabilitation, MSPSS ‘family’ was significantly positively associated with HSS among healthy women (b = 0.38, *p* = 0.019) (Table 6).

The extended model (*F*_6,102_ = 9.29, *p* < 0.001) revealed that MSPSS sustained its significant and positive association with HSS (b = 0.205, *p* < 0.001) when adjusted for KEDS (sum score), living in a relationship, number of children at home and mothers’ age. In line with mothers in rehabilitation, KEDS was found to have a significant inverse correlation with HSS, but the correlation was weaker (b =  − 0.185, *p* = 0.009) (Table 6).

## Discussion

Focus of the current study was to investigate mothers’ health from a caring science perspective taking women’s suffering into consideration. Mothers living with exhaustion and long-lasting pain reported similar health experiences and suffering as their childless counterparts, but significantly poorer health and more suffering than healthy mothers. A small difference between healthy mothers and healthy women without children was found, indicating that mothers experienced better health and less suffering than women without children. Purpose and meaning in life are usually mentioned as the great rewards of parenthood [6]. However, research in the field has come to inconclusive results of whether motherhood in itself enhances or burdens women’s overall health [26, 50]. We found low perceived social support to be a central factor as well as an equally high share of caring responsibility for relatives that united women with and without children in their suffering. The mothers living with exhaustion and long-lasting pain perceived significantly less social support from their family, friends, and significant others than healthy mothers which was related to more suffering. This underlines how vulnerable these women are on a relational level and that their suffering seems to be related to a perceived loneliness in life [14]. Among the two groups of childless women, the relationship between health and suffering and perceived social support was weaker or even non-significant. Our results suggest that mothers’ health and suffering is to a larger extent dependent on emotional and practical support when compared with the health of women without children. It has previously been emphasized how crucial it is for mothers’ well-being to be emotionally embedded in authentic relationships when they are challenged as mothers [51].

Within caring sciences, individuals are considered relational and fundamentally dependent on community [52]. There is evidence for a significant overlap between physical and social pain, suggesting that similar gene expressions, inflammatory responses and neural pathways are activated in both physical and social pain reactions [38]. This implies that women and in particular mothers need to be cared for in a way that make women feel they are emotionally supported, but also help them strengthen their immediate relationships with others. Considering the relational vulnerability of these women, health care should avoid an isolated focus on the woman and her disorder but also involve significant others in the care to alleviate suffering related to such women’s life situations.

It has previously been emphasized that social support benefits the health of individuals’ suffering when it is of high quality, arising from meaningful social connections, rather than from the mere quantity of persons providing it [38, 53]. Among various sources of perceived social support, our results indicate that support from significant others is the most important for their own health and alleviation of suffering. Mothers in rehabilitation were more often single mothers compared to the sample of healthy mothers. However, social support from various sources was still important whether the women were in a relationship or not. Being in a relationship in itself does not guarantee mothers’ well-being but unconditional comfort and support when needed, regardless of who provides the support [51]. Considering the aspect of quality and meaningful social connections, the study’s caring science approach reveals some new insights into how mothers’ health and their social environment are possibly connected. One explanation could be that an authentic interaction with family, friends and significant others might contribute to enhance their own life-understanding, hope and a meaningful integration of suffering into life. According to Rehnsfeldt and Eriksson [21], such meaning-creating encounters give opportunities to suffer on a conscious level, implying to be in a struggle between meaninglessness and meaning. In the communion with others, their own suffering might be confirmed, and space might be given to express one’s own suffering, and consequently, an individual’s suffering is alleviated and becomes bearable [21]. Both exhausted women and women with long-lasting pain reported signs of unbearable suffering that possibly could be alleviated as a result of supportive and caring encounters. Such encounters might be indispensable for mothers facing various challenges in their daily life with children [6, 51]. Our results highlight how the health of a mother depends on her immediate social ties stressing that relational aspects of women’s health need to be addressed by health care.

A reverse causality needs to be considered for the association of health and suffering with social support. Mothers suffering from exhaustion and pain might not have the energy to maintain or make use of their social support available. Such an explanation could be particularly plausible among mothers who already have high caregiving responsibilities related to parenthood limiting their personal resources to care for their social network. For example, a study on individuals undergoing rehabilitation for long-lasting pain found that these patients tend to underutilize their social support available [53]. Further, some distressed individuals might consciously avoid burdening others with their own problems and concerns [29]. But a reverse scenario could also be the case, that friends or colleagues withdraw from the social environment because they might be overwhelmed by or insecure in encountering a person living with a disorder or disability [54, 55]. When adjusting for exhaustion as a possible confounder of the association between social support and health and suffering, the association decreased, but remained significant. This indicates that the association of perceived social support and health and suffering is to a certain degree confounded by exhaustion and, thus, in parts can be explained by a lack of energy to socialize.

Regardless of the direction of possible causality, the association of social support with mothers’ health and suffering has several implications for clinical practice. Social support is a mutual interaction and not one-sided and second, not all social support is positive [29]. Consequently, mothers need to care about their social interactions with others and actively engage in relationships that improve their health and alleviate their suffering. This implies that mothers living with pain and exhaustion possibly would benefit from interventions encouraging them to form joyful and meaningful social ties making their suffering bearable [38]. Interestingly, support from family did not significantly correlate with health and suffering of mothers in rehabilitation when adjusted for support from friends and significant others, whereas the two latter ones remained significantly correlated. This suggests that women in rehabilitation already rely on social support from chosen resources (in contrast to family that cannot be chosen), but that they possibly experience a gap between support they need and perceived support. Possible reasons could be that mothers’ chosen social network is vulnerable in itself or deficient. Mothers might need help in surrounding themselves with more supportive friends and significant others according to their own needs and most beneficial for their health [38]. Another explanation could be—as previously mentioned—that individuals living with pain (and exhaustion) should learn to ask for support to a greater extent than they are used to [53]. Further, family/couple counseling or nurses or midwives following the mother and her family through a period could serve as a source of emotional support providing space to express their own suffering. Nurse-led support programs for vulnerable mothers addressing human ecology, self-efficacy and human attachment were shown to be beneficial for various mother- and children health outcomes [56].

On a societal level, the intertwinement of mothers’ health and suffering with social support might be more challenging to understand because the Swedish welfare system internationally is recognized as exemplary in regard to women’s opportunities to reconcile motherhood and work [57]. The Swedish society highly rates the values of equality, gender equality and individualism, but the other side of the coin is that it risks pushing traditional values of dependence on family and community to the sidelines [58]. The Swedish discourse in which gender equality is interwoven with individualism might create a context in which support from the immediate social environment might be undervalued. Consequently, it might risk leaving some mothers lonelier and more isolated with their social and emotional needs. Both health care and employers need to acknowledge these health risks for mothers and encourage women to create meaningful social ties. Interventions that created meaningful and authentic connections between vulnerable mothers were found to make mothers with a stressful working life feel cared for and less lonely [59].

### Limitations

A major limitation of the study is that the healthy reference sample exclusively consisted of health care professionals studying at a medical university. Thus, a comparison and an adjustment of sociodemographic confounders like age, education, and income was not considered to be meaningful. A reference sample from the general population would have been more suitable. However, other studies within the field found these sociodemographic factors to not be that influential on mothers’ health [60]. Regarding the data collected, it would have been of interest to specifically ask women whether they have children with special needs to map more specifically women’s total caregiving responsibility related to their motherhood. However, some women took the initiative to report this information in the free comment fields.

## Conclusion

Describing health and suffering from a caring science perspective widened the common medical and biopsychosocial understanding of exhaustion and long-lasting pain among women with an existential dimension of suffering and meaning in life. Women living with exhaustion and long-lasting pain are vulnerable to suffering without sufficient emotional and practical support, making their suffering unbearable. Particularly in women who are mothers, health and suffering interact with their immediate social environment on an existential level. Mothers living with exhaustion and pain need reliable social ties that create meaning in life and thereby make suffering bearable and reconcilable with health. Health care must address this intertwinement of mothers’ health, suffering and social support. Both preventive interventions on a primary care level and a specific focus in rehabilitation on social support could help mothers to actively reflect on and form social ties that are perceived as alleviating suffering and beneficial for their own health. In clinical encounters with women presenting with exhaustion or long-lasting pain, it is important to empathize and provide space for suffering as expressions of emotional support and caring.

## Data Availability

Raw data are archived at Karolinska Institutet, Sweden. Data supporting the findings of this study are available from the corresponding author AG on reasonable request.

## Abbreviations

HSS: Health and Suffering Scale
KEDS: Karolinska Exhaustion Disorder Scale
MSPSS: Multidimensional Scale of Perceived Social Support
